# Highly and Stably Water Permeable Thin Film Nanocomposite Membranes Doped with MIL-101 (Cr) Nanoparticles for Reverse Osmosis Application

**DOI:** 10.3390/ma9110870

**Published:** 2016-10-26

**Authors:** Yuan Xu, Xueli Gao, Xiaojuan Wang, Qun Wang, Zhiyong Ji, Xinyan Wang, Tao Wu, Congjie Gao

**Affiliations:** 1 Key Laboratory of Marine Chemistry Theory and Technology, Ministry of Education, Ocean University of China, Qingdao 266100, China; tcwdxy@163.com (Y.X.); safiya0524@163.com (X.W.); ouc_wangqun@163.com (Q.W.); gaocjie@ouc.edu.cn (C.G.); 2 College of Chemistry and Chemical Engineering, Ocean University of China, Qingdao 266100, China; 3 School of Marine Science and Engineering, Hebei University of Technology, Tianjin 300130, China; jizhiyong@hebut.edu.cn; 4 Shandong Zhaojin Motian Co. Ltd., Zhaoyuan 265400, China; zy_wxy@163.com; 5 Ocean College, Zhejiang University of Technology, Hangzhou 310000, China; wutao@zjut.edu.cn

**Keywords:** metal organic frameworks, MIL-101 (Cr), thin film nanocomposite, reverse osmosis, desalination, interfacial polymerization

## Abstract

A hydrophilic, hydrostable porous metal organic framework (MOF) material-MIL-101 (Cr) was successfully doped into the dense selective polyamide (PA) layer on the polysulfone (PS) ultrafiltration (UF) support to prepare a new thin film nanocomposite (TFN) membrane for water desalination. The TFN-MIL-101 (Cr) membranes were characterized by SEM, AFM, XPS, wettability measurement and reverse osmosis (RO) test. The porous structures of MIL-101 (Cr) can establish direct water channels in the dense selective PA layer for water molecules to transport through quickly, leading to the increasing water permeance of membranes. With good compatibility between MIL-101 (Cr) nanoparticles and the PA layer, the lab made TFN-MIL-101 (Cr) membranes integrated tightly and showed a high NaCl salt rejection. MIL-101 (Cr) nanoparticles increased water permeance to 2.2 L/m^2^·h·bar at 0.05 w/v % concentration, 44% higher than the undoped PA membranes; meanwhile, the NaCl rejection remained higher than 99%. This study experimentally verified the potential use of MIL-101 (Cr) in advanced TFN RO membranes, which can be used in the diversified water purification field.

## 1. Introduction

Reverse osmosis (RO) is a pressure-driven membrane separation technology, which has been rapidly developing and widely applied in seawater, brackish water and the sewage desalination process [1,2,3]. Compared with the conventional thermal-based desalination technologies, RO is more energy-efficient and can produce fresh water at a lower cost. Most commercial RO membranes have a thin film composite (TFC) structure with an ultrathin polyamide (PA) selective layer. The high cross-linked PA selective layer prepared by the interfacial polymerization process has good hydrophilicity, mechanical strength, thermal/chemical stability, selectivity, and cost advantages. However, the water permeance of PA composite membranes are slightly low due to the high extent of cross-linking [4]. Given this situation, there is still an opportunity to improve TFC RO membranes by enhancing their water permeability. Increased water permeability leads to a reduced membrane area and lessened operation pressure, which will further enhance the efficiency of the RO water treatment process [5]. A recent concept for modification of TFC membranes is incorporating porous materials into the PA layer to form a thin film nanocomposite (TFN) structure [6]. The majority of the filled porous materials are inorganic nanoparticles, such as zeolite [7,8,9,10], silica [11,12,13], and carbon nanotubes [14,15]. The porous nanoparticles can establish direct water channels in the dense selective PA layer for water molecules to transport through quickly, leading to increasing water permeance [16,17]. However, nonselective voids in conventional TFN membranes are inevitable due to the weak affinity between inorganic porous materials and organic PA layer, which might bring adverse effects to the performance and stability of RO membranes [18,19,20,21,22]. Recently, novel porous materials have been discovered to be novel membranes materials.

A novel class of porous materials, metal organic frameworks (MOFs), have attracted great attention due to their structural and functional properties, such as ultrahigh and controlled porosity, large internal surface areas, tunable pore size and type [23,24,25]. MOFs are hybrid organic–inorganic solid compounds constructed from metal containing nodes and organic linkers [26,27]. With better affinity for organic polymers owing to the organic linkers present in their structure [19,20,21], MOFs are expected to be ideal porous materials for preparing TFN RO membranes. MOFs membranes and MOFs mixed matrix membranes (MMMs) have already been applied widely in gas adsorption and separation [28,29,30,31,32,33,34,35,36]. Recent studies also have begun to focus on the application of MOFs in liquid treatment, such as organic solvent nanofiltration (OSNF) and pervaporation. Basu et al. doped MOFs [MIL-47,HKUST-1, MIL-53(Al) and ZIF-8] in polydimethylsiloxane (PDMS) membranes to reject Rose Bengal (RB) from isopropanol, the MOFs doped MMMs showed significantly higher retention of RB than undoped PDMS membranes [37]. Liu et al. prepared organophilic pervaporation membranes by adding ZIF-8 nanoparticles into silicone rubber membranes, the ZIF-8 doped membranes showed promising performance for recovering bio-alcohols from dilute aqueous solution [38]. Sorribas et al. reported that the permeate fluxes of PA/MOFs [ZIF-8, MIL-53 (Al), NH2-MIL-53 (Al) and MIL-101 (Cr)] membranes were 1.6–5.5 times higher than the pure PA membrane for the separation of styrene oligomers from methanol and tetrahydrofuran [19]. However, there is few research of MOFs doped membranes for water treatment in case of the hydration reaction involving ligand displacement and hydrolysis would destroy the topology structure and affect the properties of some MOFs (e.g., MOF-5 and HKUST-1) [39,40,41,42]. Therefore, only hydrostable MOFs materials have the potential application in water treatment.

In this work, MIL-101 (Cr) (MIL stand for Materials of Institut Lavoisier), a chromium based porous MOFs material [43], was firstly applied in manufacturing TFN RO membranes for water treatment. Compared with other water stable MOFs (e.g., ZIF-8 and UIO-66) [20,39,44], MIL-101 (Cr) possesses larger pore size and surface area, which can provide more and broader water channels. As a hydrophilic material, MIL-101 (Cr) can upgrade the surface hydrophilicity of membranes by attracting more water molecules [20]. Furthermore, most windows of the cages of MIL-101 (Cr) are pentagonal, and the channel architecture without breathing effect is expected to be unyielding during the water treatment process under the RO operation pressure [45]. Herein, we prepared MIL-101 (Cr) nanoparticle modified TFN RO membranes by the interfacial polymerization process. The influence of MIL-101 (Cr) nanoparticles dispersed phases and added amount on RO performance for rejecting NaCl salt from water were investigated. With increasing the MIL-101 (Cr) concentration, the water permeance of the TFN-MIL-101 (Cr) membranes increased and the NaCl salt rejection of the TFN-MIL-101 (Cr) membranes could maintain a high level. This study experimentally verified the potential of MIL-101 (Cr) in constructing highly and stably water permeable TFN RO membranes for water treatment.

## 2. Materials and Methods

### 2.1. Materials and Reagents

Chromium(III) nitrate nonahydrate (Sinopharm Chemical Reagent Co. Ltd., Shanghai, China), terephthalic acid (Sinopharm Chemical Reagent Co. Ltd., Shanghai, China), and methanol (Sinopharm Chemical Reagent Co. Ltd., Shanghai, China) were used to synthesize MIL-101 (Cr) nanoparticles. Trimesoyl chloride (TMC; TCI Co. Ltd., Shanghai, China), m-phenylenediamine (MPD; Sigma-Aldrich, Shanghai, China), and n-hexane (Sinopharm Chemical Reagent Co. Ltd., Shanghai, China), were used to prepare the PA layer on the polysulfone (PS) support. PS substrate was purchased from the Hangzhou Water Treatment Center (Hangzhou, China). All chemicals were of analytical grade and used without further purification.

### 2.2. Synthesis of MIL-101 (Cr)

Chromium(III) nitrate nonahydrate [Cr(NO_3_)·9H_2_O, 2.0 g, 5 mmol], terephthalic acid (0.83 g, 5 mmol), and deionized water (20 mL) were blended and briefly sonicated, resulting in a dark blue-colored suspension. The suspension was placed in a Teflon-lined autoclave and kept in an oven at 218 °C for 16 h without stirring. After the synthesis and equilibration at room temperature, the MOF solids were separated from water using a centrifuge (7000 r/min, 5 min) and washed with methanol. The resulting solids were separated by centrifugation, dried at 75 °C overnight, and then put under vacuum at ambient temperature for 2 days [46].

### 2.3. Preparation of TFC and TFN-MIL-101 (Cr) Membranes

Our strategy for in situ preparing thin film MIL-101 (Cr) nanocomposite membranes was directly adding MIL-101 (Cr) nanoparticles (0.025% to 0.1% w/v) into a 0.1 w/v % TMC hexane solution and then pouring the mixed solution onto a PS ultrafiltration support that had been immersed in a 2 w/v % MPD aqueous solution for 2 min. After 90 s of interfacial polymerization progress, a PA layer embedded with MIL-101 (Cr) nanoparticles formed on the PS ultrafiltration (UF) support. The finally prepared TFN-MIL-101 (Cr) membranes were heat cured at 120 °C for 10 min in an oven and then stored in deionized water before the performance test. TFC membranes without MIL-101 (Cr) nanoparticles and TFN-MIL-101 (Cr) membranes obtained by adding MIL-101 (Cr) nanoparticles into MPD aqueous solution were also prepared as controls.

### 2.4. Characterization Methods

The Attenuated Total Reflection Flourier Transformed Infrared (ATR-FTIR) spectroscopy was performed using a Tensor 27 spectrometer (Bruker, Karlsruhe, Germany) at room temperature. The X-ray diffraction (XRD) of the MIL-101 (Cr) was recorded on a Bruker D8 ADVANCE instrument (Bruker, Karlsruhe, Germany) equipped with a Cu Kα radiation within the range of 2*θ* = 5° to 16° at the rate of 1°/min. The nitrogen sorption isotherm was collected by a Micromeritics ASAP 2420 analyzer (Micromeritics Instrument Corporation, Norcross, GA, USA) at 77 K. A multiple point Brunauer–Emmet–Teller (BET) method was used to calculate the specific surface area of MIL-101 (Cr). Water vapor adsorption of MIL-101 (Cr) was measured with a vapor sorption analyzer (TA vti-sa, New Castle, DE, USA) at 308 K. The water absorption capacity of lab-synthesized MIL-101 (Cr) was calculated by a water adsorption experiment. Scanning electron microscopy (SEM) (Hitachi S-4800, Tokyo, Japan) was utilized to investigate the cross section and surface area of the membranes and the morphology of the MIL-101 (Cr) nanoparticles. Samples were deposited on sample holders with adhesive carbon foil and were sputtered with gold before measurement. The cross section was obtained by freezing and fracturing the membrane in liquid nitrogen. The X-ray photoelectron spectroscopy (XPS) measurement was performed on ESCALAB 250 spectrophotometer (Thermo Fisher, Waltham, MA, USA) to determine the elemental compositions of the membranes. Atomic force microscopy (AFM) images were recorded using Multimode-V microscope (Veeco, New York, NY, USA) in contact mode. Contact angle measurements were performed with a DSA100 contact angle analyzer (Kruss, Hamburg, Germany) using a sessile drop technique. 

### 2.5. RO Performance Test

**Figure 1:**
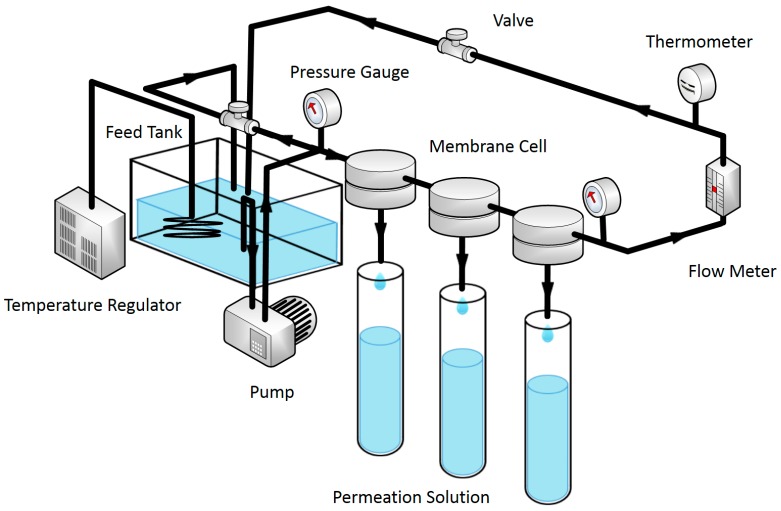
Schematic representation for the membrane performance evaluation instrument.

The RO performance of the prepared TFC and TFN-MIL-101 (Cr) membranes were characterized at room temperature. A Membrane Performance Evaluation Instrument (Figure 1) (Hangzhou Water Treatment Center, Hangzhou, China) was used to evaluate water flux and rejection of membranes via cross-flow filtration. The effective membrane area is 11.3 cm^2^ and operating pressure is 16 bar. A 2000 ppm NaCl aqueous solution was used as a feed solution. Prior to filtration, the membranes were wetted by pressurization at operating pressure for 0.5 h. Water flux (F) and solute rejection (*R*) are defined as follows:

(1) $$F=\frac{Q}{At},$$

(2) $$R\%=1-\frac{C_{p}}{C_{f}}\times 100,$$

where *Q* (L) is the volume of water passing through the membrane of surface area *A* (m^2^) during a certain time *t* (h). *Cp* and *Cf* (ppm) are the concentrations of permeate and feed solutions, respectively.

## 3. Results and Discussion

### 3.1. Characterization of MIL-101 (Cr) Nanoparticles

**Figure 2:**
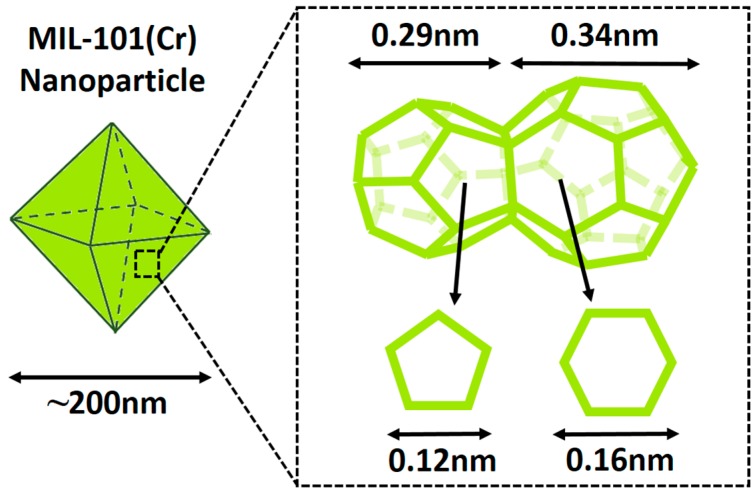
Schematic representation for the cages and openings of MIL-101 (Cr).

**Figure 3:**
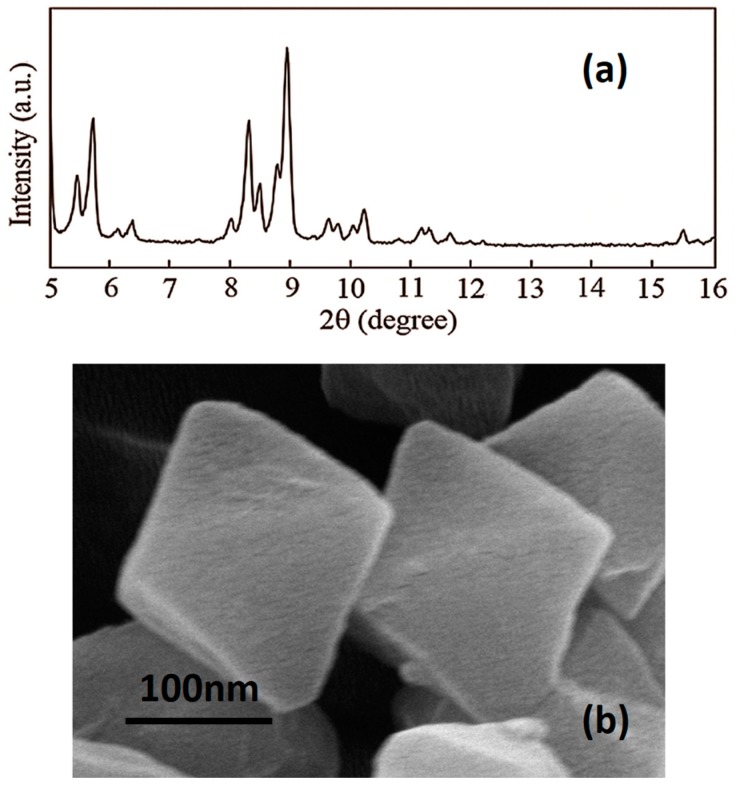
(**a**) XRD pattern of MIL-101 (Cr) nanoparticles; (**b**) SEM image of MIL-101 (Cr) nanoparticles.

MIL-101 (Cr) has a hydrophilic porous structure with 1.2 nm pentagonal/1.6 nm hexagonal openings and 2.9 nm/3.4 nm diameter cages (Figure 2) [43]. The structure of lab synthesized MIL-101 (Cr) was confirmed by XRD (Figure 3a), and the diffraction peaks agree with the reported result [46]. The SEM image of lab-made MIL-101 (Cr) nanoparticles (Figure 3b) shows the typical octahedral shapes of MIL-101 (Cr) crystals, and the nanoparticle size is around 200 nm. The similarity between MIL-101 (Cr) nanoparticle size and PA layer thickness (100 nm–300 nm) can guarantee MIL-101 (Cr) establishing longer water channels in the dense selective layer. Moreover, size matched MIL-101 (Cr) nanoparticles were expected to provide better support for the PA layer to resist the pressure induced compaction and rearrangement of the polymer chains. The BET surface area of lab-synthesized MIL-101 (Cr) was 3264 m^2^/g and the water absorption capacity of lab-synthesized MIL-101 (Cr) was 1.67 g/g. Encouraged by the high BET surface area and the high water absorption capacity, MIL-101 (Cr) nanoparticles were used to fabricate TFN RO membranes for water treatment.

### 3.2. Characterization of TFC Membranes and TFN-MIL-101 (Cr) Membranes

**Figure 4:**
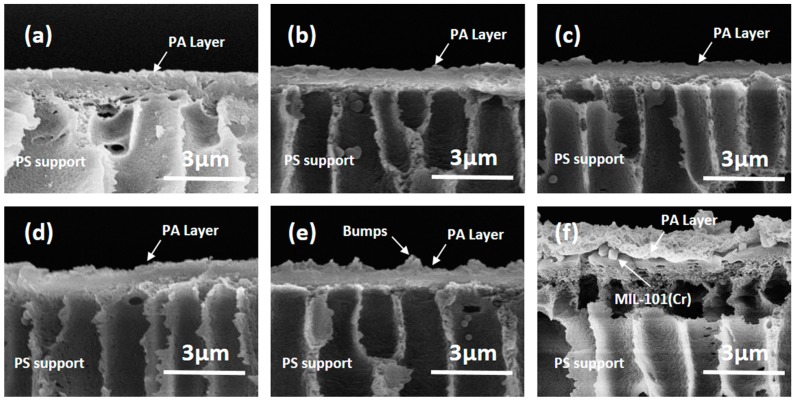
(**a**) cross section SEM image of the TFC membrane; (**b**) cross section SEM image of the TFN-MIL-101 (Cr)-O (0.025 w/v %) membrane; (**c**) cross section SEM image of the TFN-MIL-101 (Cr)-O (0.05 w/v %) membrane; (**d**) cross section SEM image of the TFN-MIL-101 (Cr)-O (0.075 w/v %) membrane; (**e**) cross section SEM image of the TFN-MIL-101 (Cr)-O (0.1 w/v %) membrane; and (**f**) cross section SEM image of the TFN-MIL-101 (Cr)-A (0.05 w/v %) membrane.

The cross section morphologies of the TFC membrane and the TFN-MIL-101 (Cr) membranes can be seen in Figure 4. TFN-MIL-101 (Cr)-O membranes prepared by adding MIL-101 (Cr) nanoparticles into organic solution (TMC hexane solution) show better integrity than TFN-MIL-101 (Cr)-A membrane prepared by adding MIL-101 (Cr) nanoparticles into aqueous solution (MPD aqueous solution). There are no clear boundaries appearing in the TFC (Figure 4a) or TFN-MIL-101 (Cr)-O (Figure 4b–e) membranes. It is unlikely that there is a clear boundary between the PA layer and the PES support of the TFN-MIL-101 (Cr)-A membrane (Figure 4f), which means a non-tight adhesion. In the interfacial polymerization process, the migration of MPD from the aqueous phase to the organic phase, which is the key step to form PA structure, was affected by MIL-101 (Cr) nanoparticles dispersed in the aqueous phase [7,13,21,47,48]. The finally formed PA layer of the TFN-MIL-101 (Cr)-A membrane was above the MIL-101 (Cr) nanoparticles (Figure 4f), which resulted in a weak combination of the PA layer, MIL-101 (Cr) nanoparticles and the PES UF support. The weak combination might bring risk to the stability of membranes for a long time under RO operation pressure, while the migration of MPD was less affected when MIL-101 (Cr) nanoparticles were dispersed in the organic phase. The finally formed PA layer can wrap MIL-101 (Cr) nanoparticles closely without visible voids (Figure 4b–e).

**Figure 5:**
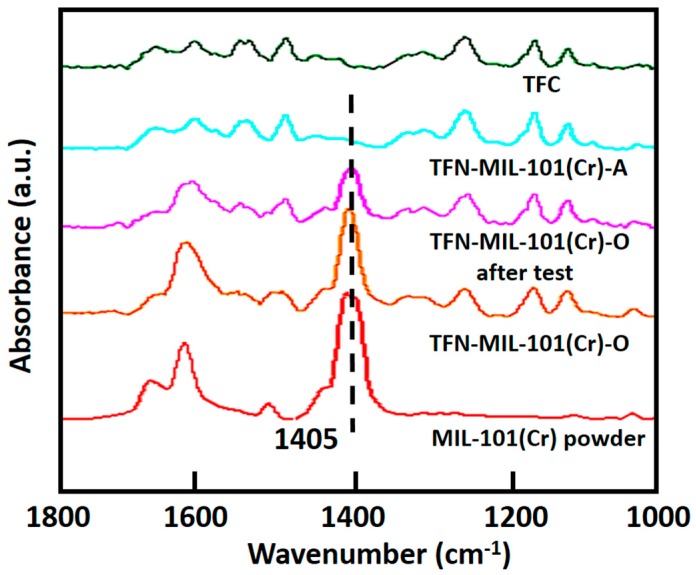
ATR-FTIR spectra of the TFC membrane, the TFN-MIL-101 (Cr)-A (0.05 w/v %) membrane, the TFN-MIL-101 (Cr)-O (0.05 w/v %) membrane, the TFN-MIL-101 (Cr)-O (0.05 w/v %) membrane after 50 h test and the MIL-101 (Cr) powder.

The ATR-FTIR spectra of the TFC membrane, the TFN-MIL-101 (Cr)-A (0.05 w/v %) membrane, the TFN-MIL-101 (Cr)-O (0.05 w/v %) membrane, the TFN-MIL-101 (Cr)-O (0.05 w/v %) membrane after 50 h test and MIL-101 (Cr) powder are shown in Figure 5. Bands between 1700 cm^−1^ and 1300 cm^−1^ correspond to *ν*(C–C), *νs*(COO), and *νas*(COO) vibrations, implying the presence of dicarboxylate linker in MIL-101 (Cr). The most intense peak (1405 cm^−1^) can be used to confirm the presence of MIL-101 (Cr) nanoparticles in the PA layer [19]. This peak appears in the spectra of the TFN-MIL-101 (Cr)-O membrane and the TFN-MIL-101 (Cr)-O membrane after 50 h test, whereas it is not present in the spectrum of the TFN-MIL-101 (Cr)-A membrane. The peak at 1405 cm^−1^ cannot be detected by depth limited ATR-FTIR in the TFN-MIL-101 (Cr)-A membrane, confirming again that MIL-101 (Cr) nanoparticles are under the PA layer, which infers that there are no through channels existing in the dense selective layer. In contrast, the peak at 1405 cm^−1^ in the spectrum of the TFN-MIL-101 (Cr)-O membrane remained about the same even after a 50 h membrane performance test, indicating that a PA-MIL-101 (Cr) structure was formed and could be maintained for a long time during the pressure-driven water treatment process. Consistent with the cross section SEM images, the ATR-FTIR test also indicates that the combination of MIL-101 (Cr) nanoparticles and PA layer is close in TFN-MIL-101 (Cr)-O membranes, which means that the organic phase is the suitable phase for MIL-101 (Cr) nanoparticle addition. Hereafter, TFN-MIL-101 (Cr) membrane characterization and performance tests will focus on TFN-MIL-101 (Cr)-O membranes.

**Figure 6:**
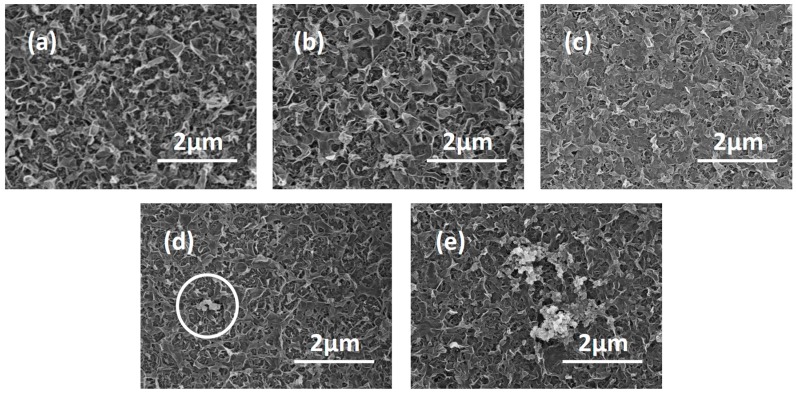
(**a**) surface SEM image of the TFC membrane; (**b**) surface SEM image of the TFN-MIL-101 (Cr)-O (0.025 w/v %) membrane; (**c**) surface SEM image of the TFN-MIL-101 (Cr)-O (0.05 w/v %) membrane; (**d**) surface SEM image of the TFN-MIL-101 (Cr)-O (0.075 w/v %) membrane; and (**e**) surface SEM image of the TFN-MIL-101 (Cr)-O (0.1 w/v %) membrane.

The morphologies of the TFC and the TFN-MIL-101 (Cr)-O membranes with adding MIL-101 (Cr) nanoparticles in the organic phase were characterized by SEM and AFM. The surface morphology of a pristine TFC membrane (Figure 6a) shows a typical “ridge and valley” structure of the dense PA layer [49]. Increasing the addition of MIL-101 (Cr) nanoparticles from 0.025 w/v % to 0.1 w/v %, surface morphologies of TFN-MIL-101 (Cr)-O (Figure 6b–e) membranes changed from the “ridge and valley” structures to smoother structures. The different morphologies of the membranes are the manifestation of the different crosslinking extent. It is widely accepted that high crosslinking extent of the dense PA layer is requisite to obtain membranes with high rejection and stability. The crosslinking extent can be reflected by the element ratios of O/N and C/N [20]. The element composition of the membranes surface (Table 1) was measured by XPS. Both O/N and C/N increase with increasing MIL-101 (Cr) loadings, which means less crosslinking extent. High concentration of MIL-101 (Cr) nanoparticles would reduce the crosslinking extent of PA layer during its forming process, so that there is a limit of the addition of MIL-101 (Cr) nanoparticles.

**Figure 7:**
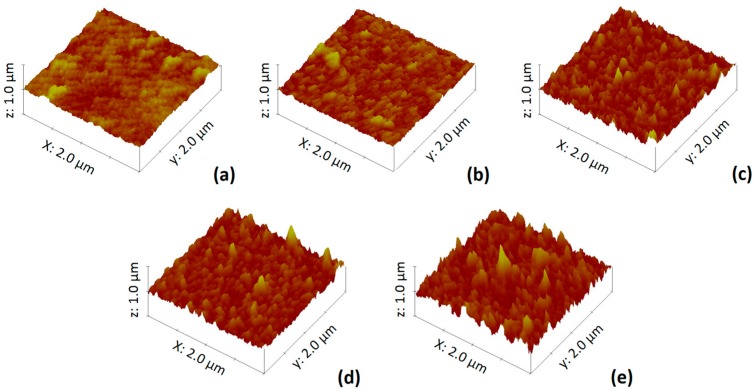
(**a**) AFM image of the TFC membrane; (**b**) AFM image of the TFN-MIL-101 (Cr)-O (0.025 w/v %) membrane; (**c**) AFM image of the TFN-MIL-101 (Cr)-O (0.05 w/v %) membrane; (**d**) AFM image of the TFN-MIL-101 (Cr)-O (0.075 w/v %) membrane; and (**e**) AFM image of the TFN-MIL-101 (Cr)-O (0.1 w/v %) membrane.

The AFM three-dimensional images of the TFC and TFN-MIL-101 (Cr)-O membranes are shown in Figure 7, and the results of roughness analysis (*Rq*) obtained from the AFM test are listed in Table 1. With the increase of MIL-101 (Cr) loading, the membrane surface became rougher and their *Rq* values increased. The roughness properties differences among these membranes were mainly due to the aggregations of MIL-101 (Cr) nanoparticles. This is consistent with the SEM images (Figure 6) of the membrane surface. At low concentration (≤0.05 w/v %), MIL-101 (Cr) nanoparticles were well-dispersed in the organic phase, most of them incorporated in situ during the interfacial polymerization and finally residing in the middle of the selective layer, so that there were no MIL-101 (Cr) nanoparticles that could be seen from the SEM images of the membrane surface (Figure 6b,c). When increasing over 0.05 w/v % MIL-101 (Cr) concentration, the aggregations of the nanoparticles, which existed in the dispersed phase and were then introduced into interfacial polymerization, were difficult to avoid. The aggregations with a large size affected the film growth and finally resided on the top of the selective layer (Figure 6d,e). Especially at 0.1 w/v % MIL-101 (Cr) concentration, more and bigger aggregations can be seen on the membrane surface (Figure 6e). The semi-exposed MIL-101 (Cr) aggregations brought bumps to the membranes surface, causing a significant increase in roughness (Table 2).

The wettability of the TFC and TFN-MIL-101 (Cr) membranes were characterized by water contact angle measurements. The results are listed in Table 2. With increasing MIL-101 (Cr) loadings, water contact angle value (*θ*) decreased. In this work, all the prepared TFC and TFN-MIL-101 (Cr)-O membranes have hydrophilic surfaces (*θ* < 90°) due to the hydrophilic carboxylic acid groups of PA and the hydrophilic hydroxyl groups of MIL-101 (Cr) [50]. As already mentioned above, with increasing MIL-101 (Cr) concentration, the crosslinking extent of PA layers decrease. The less crosslinking extent indicates that more unreacted acyl chloride groups in TMC. The unreacted acyl chloride groups then generate more carboxylic acid groups in the PA layer, which attribute to the *θ* decreasing. Moreover, the surface roughness also has influences on the wettability of membranes, and increased roughness can amplify the *θ* value decreasing. Therefore, the doped MIL-101 (Cr) nanoparticles enhanced the hydrophilic of PA membrane, which is beneficial to improving the membrane performance in the water treatment process by attracting more water molecules.

### 3.3. RO Performance

**Figure 8:**
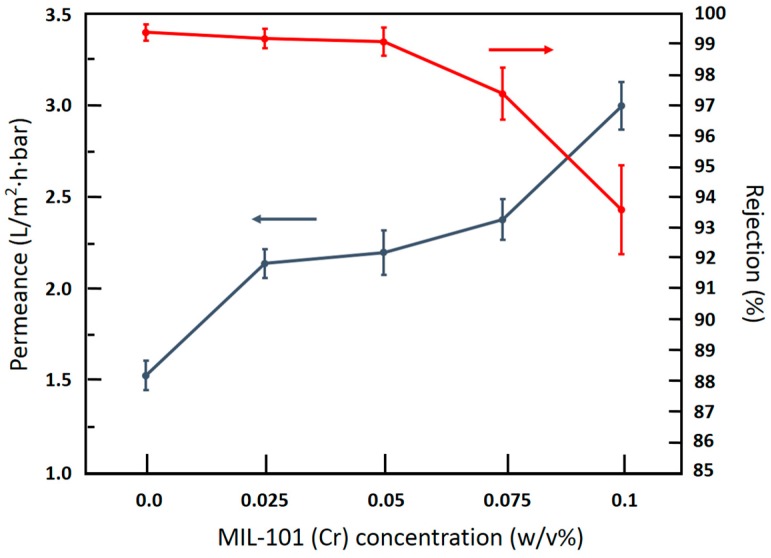
Effects of MIL-101 (Cr) concentration on water permeance and NaCl rejection of TFN-MIL-101 (Cr)-O membranes, (test conditions: 2000 ppm NaCl feed; 16 bar; 25 °C; 11.3 cm^2^ membrane area).

Figure 8 shows the effects of MIL-101 (Cr) loadings on RO water permeance and NaCl permeance of TFN-MIL-101 (Cr)-O membranes. Adding a very small amount of MIL-101 (Cr) (0.025 w/v %) increased water permeance by 40%, and the NaCl rejection of the TFN-MIL-101 (Cr)-O (0.025 w/v %) membrane maintains a high level (99.2%). At 0.05 w/v %, the water permeance of the TFN-MIL-101 (Cr)-O membrane was 44% higher than the TFC membrane and kept the NaCl rejection higher than 99%. By increasing the MIL-101 (Cr) loadings up to 0.075 w/v %, the water permeance of the TFN-MIL-101 (Cr)-O membrane was 56% higher than the TFC membrane and the NaCl rejection began to decrease (97.4%). At 0.1 w/v %, the water permeance of the TFN-MIL-101 (Cr)-O membrane was 96% higher than the TFC membrane. However, this high water permeance is relative to low NaCl rejection (93.6%). The water permeance enhancements of the TFN-MIL-101 (Cr)-O membranes are caused by a combination of the porous structure of MIL-101 (Cr), the hydrophilicity of MIL-101 (Cr) and the lower crosslinking extent of the PA structure. The micropore structure of MIL-101 (Cr) significantly contributed to the enhancement of water permeance. The typical direct channel structure provides preferential flow paths for water molecules. Water molecules can be attracted to the water paths and transport through quickly, while hydrated ions can be excluded by the MIL-101 (Cr) pores, so that the NaCl rejection can maintain a high level at a low MIL-101 (Cr) concentration (≤0.05 w/v %). At high MIL-101 (Cr) concentration, the rejection decrease was mainly caused by the aggregations of MIL-101 (Cr) nanoparticles. Although the better compatibility between MIL-101 (Cr) nanoparticles and the PA layer than traditional inorganic fillers was beneficial for avoiding nonselective voids formed in the dense PA layer, inner voids of MIL-101 (Cr) aggregations and interfacial defects between the PA and the aggregations were inevitable with increasing MIL-101 (Cr) loadings. These nonselective voids led to the NaCl rejection decrease.

Table 3 compares the TFN-MIL-101 (Cr)-O membranes in this study with other TFN membranes reported in literature. All TFN membranes give improved flux. With considering a combination of the flux and rejection, TFN-MIL-101(Cr)-O membranes exhibits better membrane performance than other TFN membranes.

**Figure 9:**
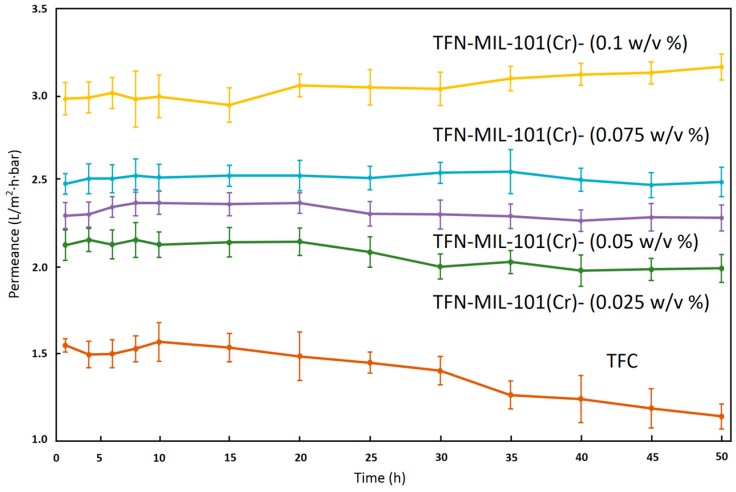
Water permeance of TFN-MIL-101 (Cr)-O membranes during 50 h stability test with 2000 ppm NaCl aqueous solution at 16 bar and 25 °C.

**Figure 10:**
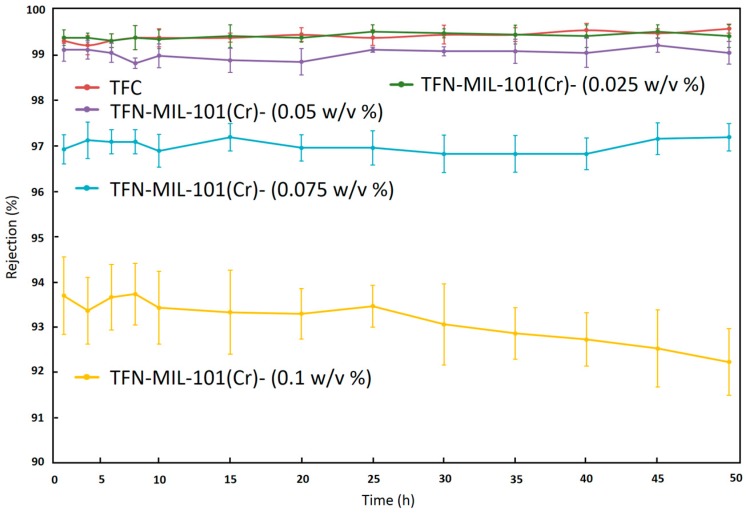
NaCl rejection of TFN-MIL-101 (Cr)-O membranes during 50 h stability test with 2000 ppm NaCl aqueous solution at 16 bar and 25 °C.

Long time (50 h) tests were used to investigate the stability of TFN-MIL-101 (Cr)-O membranes. As shown in Figure 9 and Figure 10, the separation performances of the TFN-MIL-101 (Cr)-O membranes with a small addition of MIL-101 (Cr) (≤0.075 w/v %) were stable in terms of water permeance and NaCl rejection. The TFC membrane had a 26% water permeance decline after a 50 h stability test. With the addition of MIL-101 (Cr) nanoparticles, the downward trend of water permeance is lessened. At 0.025 w/v % MIL-101 (Cr) concentration, the rate of water permeance decline of the membranes after 50 h test is 6.2%. At 0.05 w/v % and 0.075 w/v % MIL-101 (Cr) concentration, the water permeance of the membranes after 50 h test could remain the same. MIL-101 (Cr) is a hydrostable MOF material without breathing effects, and the rigid pore structure of MIL-101 (Cr) would not be damaged under the RO operation pressure in the membrane stability test process. MIL-101 (Cr) nanoparticles can play a supporting role to resist the RO operation pressure induced compaction and rearrangement of polymer chains, which leads to the stability of the water permeance. With increase of the MIL-101 (Cr) concentration up to 0.1 w/v %, the water permeance of the membrane has a 6.1% water permeance increase. In combination with the NaCl rejection results, the abnormal increase of water permeance is unstable. The NaCl rejection of the TFN-MIL-101 (Cr)-O membranes can remain stable during the 50 h stability test except for the TFN-MIL-101 (Cr)-O 0.1 w/v %. At high MIL-101 (Cr) concentration, the aggregations of MIL-101 (Cr) nanoparticles are inevitable. The aggregations may be dropped off under the high RO operation pressure during the long time test, which can bring non-selective defects to the membranes. The addition of MIL-101 (Cr) in a range of 0.025–0.075 w/v % can bring stable water performance increase to the membranes without sacrificing high NaCl rejection. The membrane selectivity cannot be improved by further increasing the MIL-101 (Cr) addition. The lower and unstable rejection at higher MIL-101 (Cr) concentration may be expected to be improved by improving MIL-101 (Cr) dispersion to avoid aggregations and defects.

## 4. Conclusions

A hydrophilic, microporous, hybrid MOF material-MIL-101 (Cr) was systematically investigated to prepare new TFN RO membranes by the interfacial polymerization method. With a good affinity for the PA dense layer owing to the organic linkers present in MIL-101 (Cr), the formed TFN-MIL-101 (Cr) membranes integrated tightly. Doped MIL-101 (Cr) nanoparticles can enhance the performance of membranes by providing direct water channels to the dense selectivity layer and changing the morphologies, roughness, crosslinking extent and wettability of membranes. At 0.05 w/v % MIL-101 (Cr) concentration, doped MIL-101 (Cr) nanoparticles increased water permeance up to 44% while maintaining NaCl salt rejection higher than 99%. With good compatibility between MIL-101 (Cr) nanoparticles and the PA layer, the increase in water permeance and the high rejection of membranes can remain stable for a long time. We believe that the new TFN-MIL-101 (Cr) RO membranes with high and stable water permeance have wide applications in the water purification field.

## Figures and Tables

**Table 1:** Summary of different prepared membranes.

Code	MIL-101 (Cr) (w/v %)	Phase for MIL-101 (Cr) Nanoparticles Addition
TFC	0	/
TFN-MIL-101 (Cr)-O	0.025	organic
	0.05	
	0.075	
	0.1	
TFN-MIL-101 (Cr)-A	0.05	aqueoous

**Table 2:** XPS result, surface roughness, water contact angle of TFC and TFN-MIL-101 (Cr)-O membranes.

MIL-101 (Cr) (w/v %)	Cr (%) ^1^	C (%) ^1^	O (%) ^1^	N (%) ^1^	C/N (-)	O/N (-)	*Rq* (nm) ^2^	*θ* (°) ^3^
0	0	76.72	13.82	9.46	8.11	1.46	47 ± 3	62 ± 2
0.025	0.04	76.69	13.93	9.34	8.21	1.49	56 ± 4	55 ± 2
0.05	0.04	76.39	14.25	9.32	8.19	1.53	58 ± 3	52 ± 2
0.075	0.07	76.65	14.11	9.17	8.36	1.54	64 ± 5	48 ± 3
0.1	0.08	76.39	14.62	8.91	8.57	1.64	72 ± 3	46 ± 2

^1^ Cr, C, O, N element atomic concentration obtained directly from XPS; ^2^ Root-mean-square surface roughness obtained from AFM, error bars based on at least three measurements; ^3^ Apparent water contact angle, error bars based on at least three measurements.

**Table 3:** Membrane performance of TFN membranes.

Filler	Concentration (w/v %)	Feed	Flux Enchancement (%) ^1^	Rejection (%)	Reference
MWNTs	0.1	PTA/water	258	98	[49]
SiO_2_	0.1	PEG600/water	121	94.7	[51]
TiO_2_	0.9	PEG1000/water	123	92.2	[52]
ZIF8	0.2	PS(400-800)/water	139	99.6	[19]
UZM5	0.02	Lubeoil/toluebe	102	96.3	[9]
MIL-101 (Cr)	0.05	NaCl/water	144	99.1	This work

^1^ The flux enhancement is defined by the flux ratio of the TFN membrane to the TFC membrane.

## Abbreviations

The following abbreviations are used in this manuscript:

RO	Reverse Osmosis
TFC	Thin Film Composite
PA	Polyamide
TFN	Thin Film Nanocomposite
MOFs	Metal Organic Frameworks
MMMs	Mixed Matrix Membranes
NF	Nanofiltration
PDMS	Polydimethylsiloxane
RB	Rose Bengal
XRD	X-ray Diffraction
SEM	Scanning Electron Microscopy
BET	Brunauer–Emmet–Teller
ATR-FTIR	Attenuated Total Reflection Flourier Transformed Infrared
XPS	X-ray Photoelectron Spectroscopy
AFM	Atomic Force Microscope
TMC	Trimesoyl Chloride
MPD	M-phenylenediamine
PS	Polysulfone
UF	Ultrafiltration
